# Recent Progress in European Advanced Therapy Medicinal Products and Beyond

**DOI:** 10.3389/fbioe.2018.00130

**Published:** 2018-09-21

**Authors:** Tracy T. L. Yu, Pravesh Gupta, Vincent Ronfard, Alain A. Vertès, Yves Bayon

**Affiliations:** ^1^Centre for Craniofacial and Regenerative Biology, King's College London, London, United Kingdom; ^2^NxR Biotechnologies GmbH, Basel, Switzerland; ^3^Tissue Engineering & Regenerative Medicine International Society European Chapter – Business Plan Competition Organising Committee, London, United Kingdom; ^4^UNT System College of Pharmacy, Fort Worth, TX, United States; ^5^Medtronic-Sofradim Production, Trévoux, France

**Keywords:** advanced therapy medicinal products, regenerative medicine, immuno-oncology, market approval, clinical translation, economic evaluation, reimbursement

## Abstract

Cell- and gene-based therapies form one of the pillars of regenerative medicine. They have the potential to transform quality of life and improve the health status of patients with genetic and cellular defects, including genetic diseases, neurodegenerative diseases and tissue malignancies, amongst others. Despite numerous challenges, in the last decade, tremendous unified efforts by research and clinical scientists in academic, translational and industry settings have resulted in tangible outcomes in the form of many marketing authorizations and approved commercial firsts, such as Glybera®, Kymriah®, YESCARTA®, Holoclar®, and Luxturna™. This report presents a succinct analysis of developments in the regenerative medicine landscape, including immuno-oncology, with a focus on the European Union and examples of clinical and commercial successes and failures. The factors that led to these exciting developments in immune-oncology are also considered. Concurrently, several key issues, spanning from the identification of unmet clinical need, associated challenges, economic evaluation to policy improvements are emphasized. Furthermore, industry insights encompassing the five-dimensional research and development framework for the focused development of medicine, pricing and reimbursement issues, technology adoption and permeation of innovative advanced therapy medicinal products in the clinical set up are reflected upon, following elaborate discussions that transpired in different thematic tracks of Tissue Engineering & Regenerative Medicine International Society European Chapter 2017 Industry Symposium.

## Introduction

Advanced therapy medicinal products (ATMPs) are defined as medical treatments that are based on genes or cells and are intended as long-term or permanent therapeutic solutions to acute or chronic human diseases at a genetic, cellular or tissue level. Diseases that require correction at a genetic (e.g., hemophilia, Rangarajan et al., 2017), metabolic (e.g., diabetes, Handorf et al., 2015), cellular [e.g., central nervous system disorders, Parkinson's (Lindvall and Björklund, 2004)] and chronic inflammatory states [e.g., immunodeficiencies, (Kumar et al., 2016) and cancer (Dattoli, 2017)], amongst others, are the frontline targets of ATMP development, due to their significant unmet medical clinical needs and the considerable market size for disease modifying or curative treatments in these therapeutic areas. Herein, key highlights of the proceedings of Tissue Engineering & Regenerative Medicine International Society European Chapter (TERMIS-EU) 2017 Industry Symposium held in Davos are presented in the context of approved ATMPs. The key messages of the lectures are discussed, from the current state of affairs in the ATMP space including IO, to issues pertaining to unmet clinical needs, a socioeconomic focus and analysis of associated challenges in current research and development practices.

## Developmental landscape of ATMPs: highlights from the TERMIS-EU 2017 industry symposium

### Successes and failures of ATMPs

**Figure 1:**
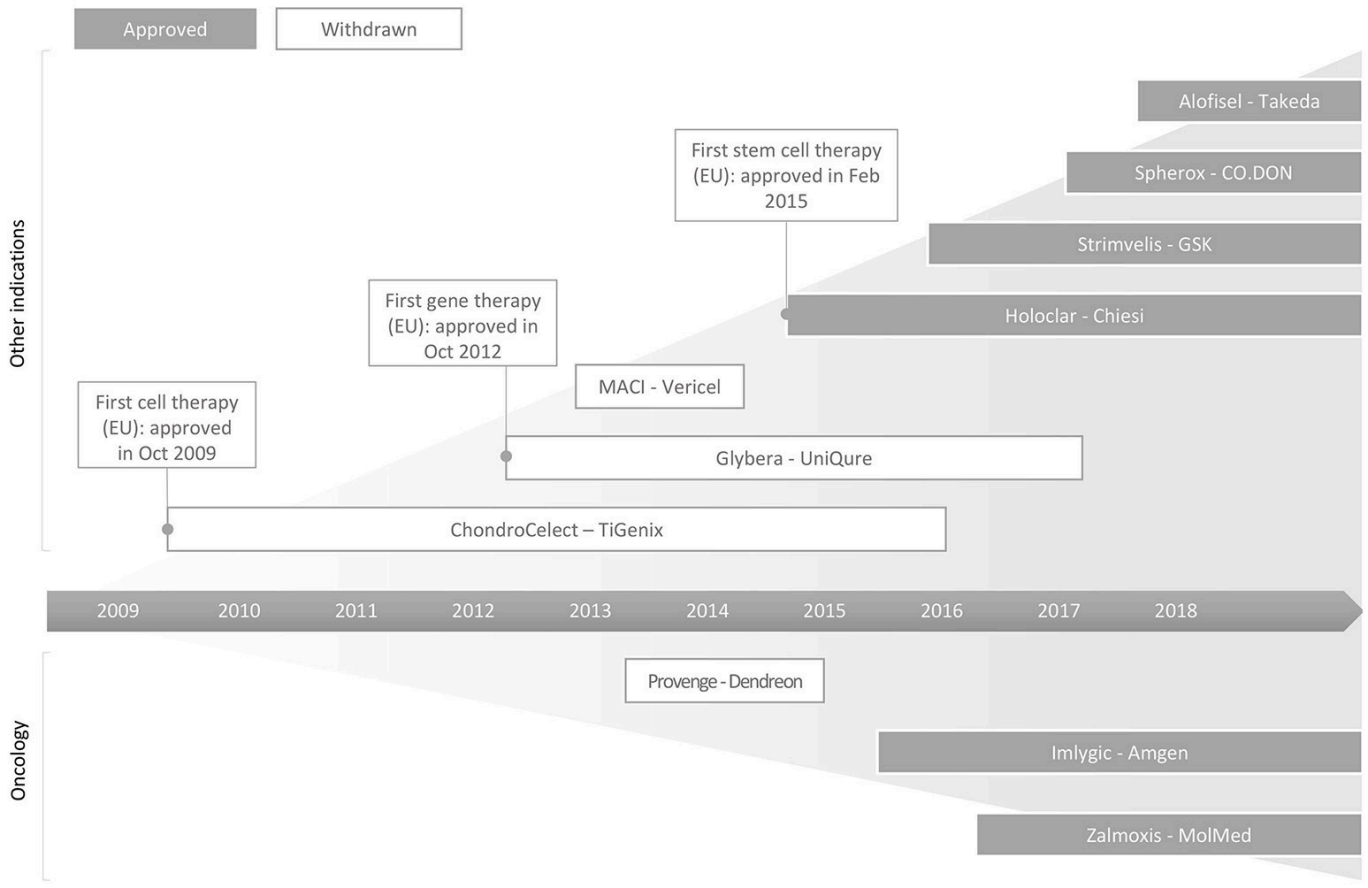
Schematic illustration of successes and failures in the commercialisation of ATMPs in the EU. As of March 2018, 10 products have received MA in the EU with Chondrocelect (TiGenix, Gelgium), Glybera (UniQure, Netherlands) and Holoclar (Chiesi, Italy) being the first approved cell, gene and stem cell therapies, respectively. Currently, six out of these 10 approved products continue to remain on the market. More information about these products, including ATMP subcategory, indication, company and date of granted marketing approval, are summarized in Table S1.

ATMPs are broadly classified into gene therapy medicinal products (GTMP), somatic cell therapy medicinal products (sCTMP), tissue engineered products (TEP), or combined ATMPs. Since the enforcement of ATMP regulation by the European Medicines Agency (EMA) in 2009 until March 2018, 10 out of 19 submitted ATMP products (Table S1) have been approved by the EMA for marketing (Abou-El-Enein et al., 2016; MolMed, 2016; European Medicines Agency, 2017; Takeda, 2018). The past decade has witnessed many commercial firsts in the history of ATMP development (Figure 1), as exemplified herewith. In October 2012, Glybera®, the first gene therapy, received marketing approval (MA) in Europe. This was developed by UniQure (Amsterdam, Netherlands) and licensed to Chiesi Farmeceutici S.p.A. (Parma, Italy). This was followed by the first commercial stem cell therapy; Holoclar®, for treatment of limbal stem cell deficiency (blindness) caused by burns (European Medicines Agency, 2014; Abbott, 2015). In 2016, GlaxoSmithKline's (GSK, London, UK) Strimvelis™, the first *ex vivo* stem cell gene therapy of its kind, was granted MA by EMA for the treatment of the very rare inherited disorder adenosine deaminase-deficient severe combined immunodeficiency (GSK, 2016; Aiuti et al., 2017). Its high market introduction price of €594,000 (NICE, 2018) per treatment was notably counterbalanced by a money back guarantee, to follow a value-based pricing rationale (Staton, 2016). As challenging as their generally high price points are, the timeline for ATMP development is also very long. For instance, Strimvelis™ took nearly 15 years since the onset of the preclinical *in vivo* study (Ferrari et al., 1991) before its developer Fondazione Telethon (Italy) received orphan designation status from the European Commission in 2005. Over a span of 25 years, multiple stakeholders had been lending their efforts to bringing this therapy from a proof-of-concept preclinical study to the granting of its MA (Candotti et al., 2012; Aiuti et al., 2017). Another example is the first chimeric antigen receptor T-cell (CAR-T) therapy, Kymriah® (tisagenlecleucel) (formerly CTL019). It was approved by the U.S. Food & Drug Administration (FDA) in August 2017 as a first-in-class therapy for the treatment of patients up to 25 years of age with refractory B-cell precursor acute lymphoblastic leukemia, or in second or late relapse. This treatment opportunity has been recently extended to relapsed or refractory large or diffused B-cell lymphoma (Daniel, 2018). Similar to Strimvelis, the development of Kymriah® took almost 30 years since the concept of redirecting T cells' potential to kill cancerous cells was introduced by Zelig Eshhar in the 1980s (Eshhar, 1997). Such developmental timelines that typically span two or even three decades from concept introduction to commercialization are one of the most common challenges for these types of products which are underlined by radical innovation (Ledley, 2018).

Another major challenge that faces most of the current ATMPs is high development and production cost which has led to pricing and reimbursement issues. Glybera® (the fourth approved ATMP in the EU) was a gene therapy product for treatment of lipoprotein lipase deficiency, despite being therapeutically successful did not set an appropriate precedent for advanced medicines due to a lack of foresight to the prospective market size, pricing, revenues, and a model of reimbursement (GlobeNewswire, 2017). Consequently, in April 2017, Glybera® at a price of $1 m per treatment, was pulled from market (Sagonowsky, 2017b). Numerically, for approximately 700 patients in Europe, a price of approximately $1 m per treatment would generate a gross revenue of $700 m (Sandle, 2012; Touchot and Flume, 2017). Assuming, 100% market penetration and a current cost of developing an ATMP of approximately $1 bn, the exit of Glybera from the European market was more an inevitability than a surprise. Even though Strimvelis was in-licensed by Orchard Therapeutics (London, UK) from GSK in April 2018 (GSK, 2018) against 19.9% equity stake, royalties and related commercial milestones payments, and Kymriah® developed by Novartis (Basel, Switzerland) passed the threshold of commercial firsts, their commercial successes remain to be assessed. Similarly, on the immuno-oncology (IO) front, an advanced prostate cancer immunotherapeutic, Provenge (sipuleucel-T), has been another commercial failure owing to pricing, reimbursement issues and likely competition from conventional therapies such as Zytiga (abiraterone acetate), which offers similar clinical benefits. Zytiga was approved in April 2011 for late stage prostate cancer, about one year after Provenge's approval in April 2010 (Dattoli, 2017). Its use was notably extended to combination with Prenisone in February 2018 for earlier forms of metastatic prostate cancer (Abou-El-Enein et al., 2016).

In addition to these economic factors, the low level of commercialization of ATMPs has also been attributed to a variety of other factors, such as the complexity of the technologies, difficulties in manufacturing processes and regulatory barriers. Several cases of ATMP clinical trial failures at advanced stage Phase III can be illustrated by examples such as HP-802-247, Fistula Advanced Therapy Trial 1 (FATT1) and Cerepro. Smith and Nephew-curated HP-802-247, a spray comprising human living cells to support healing of leg ulcers, failed in Phase III, despite promise being shown in Phase I and II studies. The most likely cause for variable and inconsistent clinical responses was due to changes in cell (primarily keratinocyte) phenotype that led to batch variabilities in cell banks (Hirschler, 2014; Kirsner et al., 2016). FATT1, a Cellerix-sponsored study to offer an autologous adipose-derived stem cell-mediated treatment option for complex cryptoglandular perianal fistulas, failed to re-establish the therapeutic benefits witnessed during Phases I and II. Multiple hurdles have been associated with this failure, such as patient inclusion criteria (severity of disease), surgical procedures, cell processing and formulations (with or without fibrin glue) employed. Even variabilities in the experience and training of the surgeons (linked to proper use of the cells) involved during the trials contributed to the dismal results (Herreros et al., 2012; Edison, 2015). Cerepro (sitimagene ceradenovec), an adenoviral gene therapy for treatment of malignant glioma developed by Ark Therapeutics, also failed to confirm therapeutic benefits in Phase III. The EMA rejected the marketing authorization application of Cerepro for two reasons: statistically underpowered clinical trial data and insufficient efficacy in extending survival time or delaying re-intervention (Mitchell, 2010). Avotermin, a human TGFβ3 recombinant formulation—despite being an example of a biologic that directs regeneration and prevents fibrosis with inherent anti-scarring attributes—also met with a similar unfortunate end. Despite showing significant therapeutic efficacy in Phase I & II, clinical results in Phase III could not be recapitulated (Occleston et al., 2011; Finnson et al., 2013). A plausible explanation could be either limited understanding of the mechanisms of action or clinical design failure. Therefore, appropriate redressal mechanisms need to be accordingly formulated and adhered, which should be derived from critical assessment of the various factors that led to failures, not only in ATMPs, but also other drug development pipelines, such as Avotermin, a well-documented case in the literature. Lessons learnt from such paralleled efforts can accelerate the pace of upcoming ATMPs' developmental life cycle. To this end, an element of response is, of course, the streamlined regulatory processes exemplified by the Twenty-first Century Cures Act (USA), passed in December 2016 to answer burning requests from patient advocacy groups for products that are intended to treat, modify, reverse or cure a serious or life-threatening disease or condition, and for which there is preliminary clinical evidence indicating the potential to address those unmet medical needs.

Contemplating the progress of ATMP technology in the last decade, a major trend that appears is that IO-based ATMPs represent a successful translation from discovery phase to the demonstration of clinical benefits to patients, relating to improved quality of life or survival.

### Focus on immuno-oncology: a tale of success

The general conceptual seeds of IO were sown many decades ago in the mid-nineteenth century by Rudolf Virchow, owing to his observations of immune cell infiltration in human tumors (Virchow, 1862). Three decades later, in 1891, William Coley demonstrated the shrinkage of osteosarcoma by injecting *Streptococcus*, highlighting the importance of immunotherapy in cancer (Coley, 1991). Almost a century later in 1988, Rosenberg's group pioneered the development of tumor specific T-cells wherein re-implanted, *ex vivo*-expanded tumor-infiltrating lymphocytes (TILs) resulted in complete responses in less than 10% of the melanoma patients. Continued global efforts have revolutionized the field of cancer immunotherapy in the past decade, with the advent of multiple modalities in advanced stage clinical trials with the three pillars of IO lead by checkpoint inhibitors, chimeric antigen receptor engineered T-cells (CAR-T, also referred to as T-bodies in earlier publications) and more recently, neoantigen-based vaccines. Since the approval of Ipilimumab in 2011, a checkpoint inhibitor targeting CTLA-4 to treat advanced melanoma patients, 26 immunotherapies have been approved for 17 different cancer subtypes. The enthusiasm in this field is reflected by major investments and 940 IO agents being in clinical development, with 1,064 in preclinical phase (Kamta et al., 2017). This excitement is rallied on the premise of ~20–30% response rate to checkpoint blockade therapies to a wide range of cancers and ~80–90% response rate to CAR-T therapies against blood cancers.

### Redundancy of immuno-oncology approaches

The success of immunotherapy as a new paradigm has notably been largely attributed to relaxed clinical trial protocols that take into account the urgent need for interventions to improve survival of cancer patients that have exhausted all options of treatment, a criterion of eligibility for immunotherapy trials. The concentration of the clinical trials on very few targets is exemplified by the fact that 164 agents target the PD1/PDL1 pathway, suggesting dramatic redundancy of strategies that might have congruent non-significant outcomes (Tang et al., 2018). Similarly, of more than 200 CAR-T cell-based clinical trials, the majority are focused on CD19-CARs to treat lymphomas, further highlighting this redundancy (Hartmann et al., 2017). Nonetheless, despite initial success in treating certain types of liquid cancers, the industry needs to move cautiously on this front as there have be numerous precedents of drug failure at Phase III. This is due to poorly understood mechanisms of action, which resulted in inappropriate side effects in a significant portion of the patient population, or limited efficacy (Jain et al., 2006; Arrowsmith and Miller, 2013).

### Challenges in immuno-oncology

Despite tremendous leaps in IO treatment regimens, solutions to challenges such as the identification and stratification of responder and non-responder patients to different IO variants or combinations remain elusive. Efforts to discover prognostic biomarkers, their validation and standardization across different cancer subtypes and to immunoscore and differentiate immunologically active and dormant tumors would result in more reliable data interpretation. The thrust of the clinical trials should not only focus on primary or secondary endpoints, but also on continuously monitoring the immune activity to assess real-time immunological perturbations that potentially can uncover mechanisms of action of therapeutic interventions. This is a critical step toward developing the next wave of rationally-designed combination cancer immunotherapies, based on a deep understanding of the mechanisms by which combination therapies influence the battle between a patient's immune system's capabilities to fight cancer, as well as the undergoing immune-suppressive processes in the tumor microenvironment that promote tumor growth (Hegde et al., 2016).

### Policy improvements to support ATMP development

Greater clarity and harmonization in ATMP regulations are needed to promote their development and commercialization. The EMA's PRIority MEdicines (PRIME, 2017) and FDA's Breakthrough Designation and Regenerative Medicine Advanced Therapy Designation (2017) have been created to support such effort. Recently, EMA published updated procedural advice to clarify the regulatory process for ATMPs with three objectives: firstly, to streamline procedural aspects and strengthen collaboration between EMA's scientific committees; secondly, to help with specific needs of ATMP developers in the evaluation procedure for initial marketing authorization; and lastly, to help navigate the regulatory process in the EU and to provide more time if necessary to submit regulatory dossiers (EMA, 2018).

Built on the early success of pioneering therapies (Table S1), three more ATMPs were approved in 2017: LUXTURNA gene therapy (Spark Therapeutics, Philadelphia, PA, USA, by the EMA), Yescarta CAR T-cell therapy (Gilead/Kite Pharma, Foster City, CA, USA, by the FDA) and Kymriah® CAR T-cell therapy (Novartis, by the EMA). More recently (March 2018), Alofisel®, an allogeneic stem cell therapy developed by TiGenix (Brussels, Belgium), was licensed to the Japanese pharmaceutical company Takeda and in the same month received marketing authorization approval in Europe for the treatment of complex perianal fistulas in Crohn's disease (Takeda, 2018).

Nevertheless, despite various milestones and setbacks, the healthcare industry is moving very fast, as evidenced by the existence of 946 clinical trials worldwide related to ATMPs, including 314 Phase I, 550 in Phase II, and 82 in Phase III, with a year-on-year growth rate of 16, 18, and 24%, respectively, as of 2017 (data from Alliance for Regenerative Medicine, January 2018). Despite all these promises, key obstacles still remain, especially regarding the reimbursement of approved ATMP therapies.

Hereon, based on discussions at the proceedings of TERMIS-EU 2017 Industry Symposium, are discussed the lessons learned in the context of unmet clinical needs, clinical development pipelines, industry insights, and related issues over the past 30 years.

## ATMP development: the way forward

### Identification of unmet clinical needs

The development of novel medicines has to fulfill unmet clinical needs or to create alternative solutions that provide a higher incremental cost-effectiveness ratio, defined as the ratio between the change in costs and change in effects (Rutigliano, 1995). Considering the timescale for the discovery of new medicines, the latter needs to prove substantial and obviously measurable improvements in health outcome in order to gain access to patients. According to Rogers, the diffusion of novel technologies typically follows an S-curve market penetration pattern and eventually reaches saturation level (Rogers, 2010). Building on several early clinical successes (Abou-El-Enein et al., 2016), ATMP development only recently progressed through the early adoption stage.

### Economic evaluation of therapies in development

Currently, high prices are still closely associated with ATMP products, due to their high cost of development, production, product storage and transportation (Bouchie, 2002). Learning from Glybera®'s record-breaking pricing approach, coincidentally the prices of the two cancer gene therapies Yescarta® and Kymriah® have been set at marginally lower price points- €316,000 and $475,000 (€402,000), respectively, which very likely have benefited from larger populations of eligible patients compared to Glybera®. If upcoming new therapies were to target larger patient populations, both their costs and prices would inevitably continue to decrease, thereby maintaining both the incentive to invest in new therapies and the affordability of such paradigm-changing therapies to payers and patients respectively. Beyond the domain of gene therapies, similar trends have also been observed in the cell therapy arena, for example Alofisel® is priced between $60,000 and $120,000 per dose and may potentially address as many as 37,000–70,000 patients worldwide (Takeda, 2018).

Simultaneous application of health economic evaluation tools and comparative analysis would ensure prioritization of those technologies that are most likely to offer higher cost effectiveness (expressed as cost per unit of health outcome) or incremental cost-effectiveness ratio (expressed as cost per quality-adjusted life-year gained) (Cosh et al., 2007). Further, early estimation of the cost and effectiveness between the new therapy and current practices and consequent revision in accordance with the emergence of new data can guide decision-making processes, technology development and resource allocation (including human resource and capital investment). Several tools and frameworks have since been developed to facilitate such processes, as exemplified by the headroom analysis method (Sculpher et al., 1997; Cosh et al., 2007). Headroom is defined as the maximum additional cost of a new treatment over the current gold standard treatment for the new treatment to be deemed cost-effective (Cosh et al., 2007). McAteer et al. have demonstrated two applications of such tools for evaluating tissue engineering products. In brief, in the case of engineered urethral tissue, the headroom was estimated at £186 (McAteer et al., 2007). At this relatively very low benefit margin, it becomes impossible to justify the cost of producing such products that are populated with viable cells. In contrast, a tissue engineered bladder, for example as a substitution organ after cancer resection, has a calculated headroom of £16,000. However, considering the limited patient numbers, the headroom benefit may still be not enough to provide adequate overall return, unless quality-of-life parameters are also factored in Cosh et al. (2007).

The orphan disease arena has long been dominated by biotechnology enterprises, partially due to a lack of competition from large pharmaceutical companies during the period between 1995 and 2015, when the blockbuster business model was still intact. Since then, large pharmaceutical firms have considerably reorganized their drug development approaches, for example deprioritising conventional therapeutic areas, such as neurology or cardiovascular, and seeking growth in specialty drugs and biologics, as well as in emerging markets, which even includes novel therapeutic targets, such as orphan diseases (Gautam and Pan, 2016). One of the most successful cases in the area of orphan diseases has been brought by the pioneer biotechnology company Genzyme (Cambridge, MA, USA), now part of Sanofi (Paris, France). Among Genzyme's portfolio, Cerezyme® became the first-line treatment for Type 1 Gaucher disease. At an annual treatment cost of $300,000 per patient, Cerezyme® alone generated $719.6 m in sales for Genzyme in 2010 (GENZYME, 2011). Though the margins will inevitably erode over time as competing therapies become available, with the right opportunity and adequate resources, meeting the break-even point is foreseeable. This is despite high development cost of $2.9 billion in 2013, for example (DiMasi et al., 2016), especially at a time of expanding awareness and evolving policy environments (Borski, 2015). Several of the world's largest pharmaceutical companies joined the rare disease arena in the early 2010s, including Novartis and Pfizer (New York, NY, USA) (Shaffer, 2010), although GSK recently U-turned from its established rare disease portfolio (Sagonowsky, 2017a; GSK, 2018).

### Industry insight on ATMP development

AstraZeneca's (Cambridge, UK) retrospective analysis from the drug pipeline development and failures during different phases of clinical trials have led them to unearth a five-dimensional framework for focused R&D of healthcare medicines. Symbolized as “the five R's,” this concept incorporates notions regarding the right target, the right tissue, the right safety, the right patients and the right commercial potential (Cook et al., 2014), which can be carefully considered before committing and investing several billion dollars for a more than decade-long R&D process. On a similar note, Merck's (Darmstadt, Germany) Translational Medicine Guide improved its R&D workflow in terms of learning, strategy, costs and performance (Dolgos et al., 2016).

Progressive iteration of the science and reproducibility of knowledge output should be strictly assessed to lay down strong principles, paving the way from proof-of-concepts to achieving translational avenues. However, this product development path is very complex. It comprises not only innovative scientific approaches, successful transition from bench solution to clinical grade product, appropriate manufacturing processes ensuring stability and quality during preparation and transportation to clinical trial sites, but also identification of eligible patients, availability of trained and experienced personnel administering therapy and rigorous follow up assessment procedures. The level of this complexity is further illustrated by the lessons learnt from the disparity of outcome between preclinical research and human translational studies, especially at phase III mesenchymal stem cell (MSC)-based therapies, but also other cell therapies, including IO products. In addition to the consideration of the tissue source of cells, the culture methods and expansion levels, differences in cell preparation, fitness, the timing of cell transfusion and functionality also have been shown to greatly contribute to clinical successes. Though many disease indications still lack robust predictive biomarkers, insights from early-phase trials may inform rational patient stratification. A noteworthy demonstration is the Mesoblast (Austria)-sponsored improved phase III clinical trial using MSCs for pediatric graft-versus-host disease (NCT02336230). The improved trial design was a consequence of rationale patient stratification contrary to the original unsuccessful trial (NCT00366145), which catered to all age groups ranging from 6 months to 70 years (Galipeau and Sensébé, 2018). The value of precise stratification of patients and recruitment at advanced stages of clinical trials has already been firmly established, resulting notably in the rescue of failed trials (Domenyuk et al., 2018). Alofisel® (darvadstrocel) has set a model of iterative strategy that enabled MA. After its former sponsor Cellerix was acquired by TiGenix, lessons learnt from Cx401 (autologous cell therapy, sponsored by Cellerix) Phase III failure had resulted in the improved Cx601 (allogeneic cell therapy, sponsored by TiGenix) Phase III trial design to ensure strict adherence to treatment protocol guidelines and ultimately brought the eureka moment to TiGenix (Edison, 2015; Panés et al., 2016; TiGenix, 2018). Furthermore, clinical trial designs should not only focus on evaluating the safety and efficacy parameters, but also better understanding the underlying pathobiology of diseases of interest by using high dimensional omics approaches, as a deeper knowledge would improve the product design steps. Such an iterative strategy could significantly reduce the odds of failures at a very advanced stage of product development, involving large patient cohorts. In addition, such research-based approaches on patients enrolled in early stages of clinical trials would allow better understanding of the underlying reasons for patients' response or non-response, which may be due to differential age, health, immune or metabolic or genetic status.

## Perspectives

Outstanding progress have been achieved in recent years in cell- and gene-based therapies, exemplified by the approval of paradigm-changing, disease-modifying or even curative treatments in many therapeutic areas, including oncology, inflammation and sensory losses (notably ophthalmology). A solid foundation for the emerging ATMP field has been cemented via the approval of the first gene- and cell-based therapies. Numerous challenges, however, remain to make these new pharmaceutical modalities permeate healthcare as deeply as monoclonal antibodies have achieved since the beginning of the 2010s. It took more than three decades for the market of products derived from monoclonal antibody technologies to become primary drivers of the pharmaceutical market. Monoclonal antibodies have revolutionized the treatment regimens of numerous diseases, achieving extraordinary breakthroughs, most notably in the inflammation and oncology therapeutic areas, thereby significantly contributing to patient fitness, prolonged survival, and enhanced quality of life. The next revolution in healthcare is undoubtedly that of regenerative medicine, based on the realization of the potential of genes and living cells as drugs. As highlighted in the present article, initial progress has already been made, with patients achieving, for example, complete remission in acute lymphoblastic leukemia, despite having an otherwise extremely poor prognosis, or reversing the devastating effects on vision of chemical burns or other types of corneal injury. The EMA has been one of the main actors within the sector, making several important first approvals. Nonetheless, beyond complex technical and translational issues, numerous challenges remain that have not been discussed here. It is also very important to address those other concerns efficiently.

Firstly, it would be preferable if a smoother pathway than the one that practitioners of the monoclonal antibody field had to take before their disruptive technologies and products could be adopted by the industry and thus permeate widely into the healthcare system. Technology adoption by large pharmaceutical companies and the diffusion of new ATMP technologies remains slow, in spite of intense interest in CAR-T cell products and several notable transactions in the field of MSC therapeutics.

Secondly, innovative reimbursement models must be implemented, as the current model is clearly ill-adapted to the unique nature of some ATMP's effectiveness, e.g., as one-time treatments to cure chronic diseases, or to improve the quality of life for a prolonged period of time. Here, it is critical to maintain a cycle of investments through a balance between incentives for innovation (price of a therapy) and market access (affordability and availability). On the other hand, policy makers and regulatory agencies worldwide have already increased the pace of the new revolution within healthcare, as exemplified by the enactment and active implementation of novel regulatory routes that respond to the urgency expressed by patients and their advocates, while ensuring safety and efficacy considerations are appropriately followed.

The next frontier to complete the current transformation of the healthcare sector will be to define an effective mode to combine therapeutic modalities, either sequentially or in parallel. For example, several large pharmaceutical companies are slanting toward improving long-term disease control and survival across a range of cancers by combining different drugs, for example, coupling a checkpoint inhibitor with an established treatment regimen. The approach of combination therapies has already achieved a tremendous impact in healthcare, with oncology innovation being a major driver of scientific, technical and operational progress.

## Author contributions

YB initiated the paper. TY and PG wrote the manuscript with input from all authors. VR, AV and YB revised the manuscript critically for important intellectual content. All authors read and approved the final manuscript.

### Conflict of interest statement

YB is an employee of Medtronic but was not restricted in any way. VR is employed by Cutiss and HairClone. The remaining authors declare that the research was conducted in the absence of any commercial or financial relationships that could be construed as a potential conflict of interest.
