# Supplementary material for: Recent Progress in European Advanced Therapy Medicinal Products and Beyond
Source: Front Bioeng Biotechnol. 2018 Sep 21;6:130. doi: 10.3389/fbioe.2018.00130 (PMC6161540; doi:10.3389/fbioe.2018.00130)
Supplement: Supplementary file 1 [file Table_1.DOCX]

Supplementary Material

Recent progress in European advanced therapy medicinal products and beyond

Tracy T. L. Yu, Pravesh Gupta, Vincent Ronfard, Alain A. Vertès, Yves Bayon*

*** Correspondence:** Yves Bayon: yves.bayon@medtronic.com

Table S1. Summary of ATMPs obtained marketing authorization in the EU (as of March 2018), related to Figure 1

| **Product** | **ATMPs Subcategory** | **Indication** | **Company** | **Date of Granted Market Authorisation (MA)** |
| --- | --- | --- | --- | --- |
| Alosifel | CTMP | Rectal Fistula | Takeda Pharma A/S (Denmark) | March 23, 2018 |
| Spherox | TEP | Used in adults to repair cartilage in the femoral condyle and the patella (knee cap), where the size of the affected area is no larger than 10 cm² | CO.DON AG (Germany) | July 10, 2017 |
| Zalmoxis | GTMP | Adjunctive treatment in haploidentical haematopoietic stem cell transplantation (HSCT) of adult patients with high-risk haematological malignancies | MolMed (Italy) | August 18, 2016 |
| Strimvelis | GTMP | Treatment of patients with severe combined immunodeficiency due to adenosine deaminase deficiency (ADA-SCID) | GSK (UK) | May 26, 2016 |
| Imlygic | GTMP | Imlygic is an oncolytic immunotherapy used to treat adults with melanoma | Amgen Europe BV (Netherlands) | December 16, 2015 |
| Holoclar | TEP | Stem cells transplantation for corneal diseases | Chiesi Farmaceutici S.p.A. (Italy) | February 17, 2015 |
| Provenge | sCTMP | Immunotherapy for prostate cancer | Dendreon (United States) | September 6, 2013 (MA withdrawn in May 2015) |
| MACI | TEP | An implant used to repair cartilage defects at the ends of the bones of the knee joints | Vericel Denmark ApS (Netherlands) | June 27, 2013 (MA suspended in September 2014) |
| Glybera | GTMP | Authorised for treating adults with lipoprotein lipase deficiency who has severe or multiple attacks of pancreatitis | UniQure Biopharma BV (Netherlands) | October 25, 2012 (MA ended in October 2017) |
| ChondroCelect | TEP | Used in adults to repair cartilage in the knee | TiGenix NV (Belgium) | October 5, 2009 (MA withdrawn in July 2016) |
